# ﻿Another new karst-dwelling rock gecko in the *Cnemaspissiamensis* group (Reptilia, Gekkonidae) from Kanchanaburi Province, western Thailand

**DOI:** 10.3897/zookeys.1226.138464

**Published:** 2025-02-06

**Authors:** Attapol Rujirawan, Akrachai Aksornneam, Siriporn Yodthong, Parinya Pawangkhanant, Bryan L. Stuart, Anchalee Aowphol

**Affiliations:** 1 Animal Systematics and Ecology Speciality Research Unit, Department of Zoology, Faculty of Science, Kasetsart University, Bangkok 10900, Thailand Kasetsart University Bangkok Thailand; 2 Biodiversity Center Kasetsart University (BDCKU), Bangkok 10900, Thailand Biodiversity Center Kasetsart University (BDCKU) Bangkok Thailand; 3 Department of Biological Science, Faculty of Science, Ubon Ratchathani University, Ubon Ratchathani 34190, Thailand Ubon Ratchathani University Ubon Ratchathani Thailand; 4 Rabbit in the Moon Foundation, Suan Phueng, Ratchaburi 70180, Thailand Rabbit in the Moon Foundation Ratchaburi Thailand; 5 North Carolina Museum of Natural Sciences, 11 West Jones Street, Raleigh, North Carolina 27601, USA North Carolina Museum of Natural Sciences Raleigh United States of America

**Keywords:** *Cnemaspisenneaporus*, karst formations, molecular phylogenetics, morphology, Tenasserim Mountain Range

## Abstract

A new species of the *Cnemaspissiamensis* group is described from Wang Khrachae District, Kanchanaburi Province, western Thailand based on morphological and molecular data. The new species, *Cnemaspisenneaporus* Rujirawan, Aksornneam & Aowphol, **sp. nov.**, is distinguished from other species in the *C.siamensis* group by having the combination of SVL 42.2 mm in adult male (*n* = 1), 43.7 mm in adult female (*n* = 1); eight supralabials; seven or eight infralabials; ventral scales smooth; nine continuous precloacal pores in male; 17 or 18 paravertebral tubercles linearly arranged; tubercles on lower flanks present; lateral caudal furrows present; no caudal tubercles in the lateral furrows; ventrolateral caudal tubercles present on original portion of tail; caudal tubercles not encircling tail; subcaudals smooth; no enlarged median subcaudal row; two postcloacal tubercles on each side of tail; no shield-like subtibial scales; subtibial scales smooth; no enlarged submetatarsal scales; 26–28 subdigital lamellae on the fourth toe; sexually dimorphic for dorsal and ventral colour pattern; prescapular marking absent; gular marking absent; and yellow colouration in life on all ventral surfaces of head, body and tail in adult male. Phylogenetically, the new species is recovered as the sister taxon to *C.huaseesom*, but the two species are separated by 8.3–9.4% uncorrected pairwise genetic divergences in the mitochondrial NADH dehydrogenase subunit 2 gene and flanking tRNAs.

## ﻿Introduction

The rock gecko genus *Cnemaspis* Strauch, 1887 is the second-most diverse gekkonid genus with 227 named species (Karunarathna et al. 2023; Uetz et al. 2024). *Cnemaspis* sensu stricto (= Southeast Asian *Cnemaspis*; sensu Grismer et al. 2014), hereafter *Cnemaspis*, contains at least 69 Sundaic and Indochinese species that are distributed from southern Laos, Vietnam, Cambodia, and Thailand, and southwards through Thai-Malay peninsula, Sumatra, Java, and eastwards to Borneo (Grismer et al. 2010, 2014; Riyanto et al. 2019; Ampai et al. 2022; Nashriq et al. 2022; Rujirawan et al. 2022; Kurita et al. 2024). In Thailand, *Cnemaspis* comprises 22 named species from two major clades (the Northern Sunda and Pattani clades; sensu Grismer et al. 2014). For the Northern Sunda clade, Thai *Cnemaspis* have been assigned to three species groups (the *C.affinis*, *C.chanthaburiensis*, and *C.siamensis* groups) based on morphology and molecular phylogenies (Grismer et al. 2014; Wood et al. 2017; Ampai et al. 2019, 2020, 2022; Rujirawan et al. 2022). The *siamensis* group is the most diverse species group in Thailand, comprising 16 nominal species that are distributed along the Thai-Malay Peninsula from Kanchanaburi Province in western Thailand southwards through the Isthmus of Kra region in southern Thailand (mainland and adjacent islands) and Langkawi Island, Peninsular Malaysia (Grismer and Chan 2010; Ampai et al. 2022; Rujirawan et al. 2022). Recently, *C.auriventralis* Rujirawan, Yodthong, Ampai, Termprayoon, Aksornneam, Stuart & Aowphol, 2022 was described from Erawan National Park and is closely related to *C.huaseesom* Grismer, Sumontha, Cota, Grismer, Wood, Pauwels & Kunya, 2010 from Sai Yok National Park (Grismer et al. 2010; Rujirawan et al. 2022). Both species were described from Kanchanaburi Province, western Thailand (ca 25 km between their type localities) and from similar habitat and substrate (karst associated area), but differ in elevational zonation (upland in *C.auriventralis* and lowland in *C.huaseesom*) (Grismer et al. 2014; Rujirawan et al. 2022).

**Figure 1.**
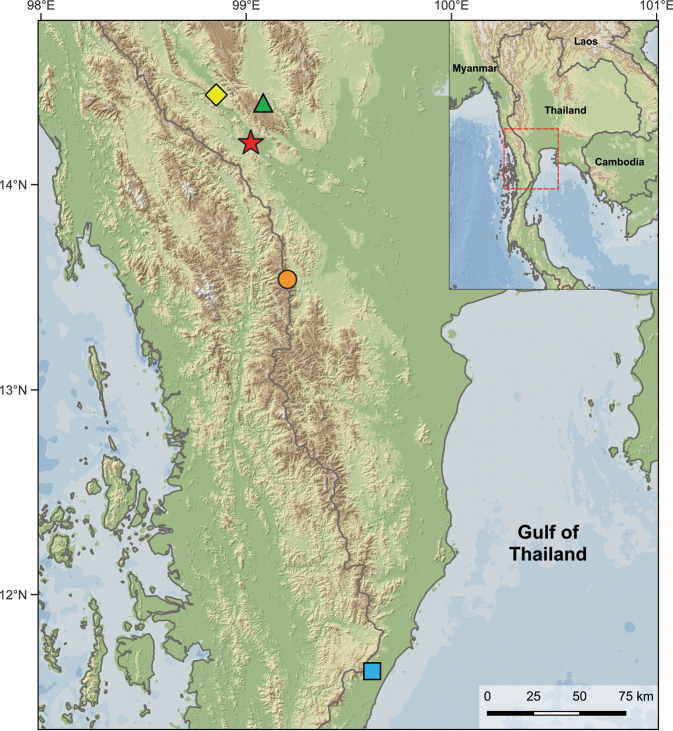
Map illustrating the type localities of *Cnemaspisenneaporus* sp. nov. (red star) at Wang Khrachae Subdistrict, Sai Yok District, Kanchanaburi Province, Thailand and its close relatives, *C.auriventralis* (green triangle), *C.huaseesom* (yellow diamond), *C.punctatonuchalis* (blue rectangle), and *C.selenolagus* (orange circle).

In November 2022, two specimens of *Cnemaspis* were collected from karst forest in Wang Khrachae Subdistrict, Sai Yok District, Kanchanaburi Province, western Thailand (Fig. 1). This population closely resembled *C.auriventralis* in colour pattern (all ventral surfaces of head, body and tail yellow in adult male) and microhabitat. Our morphological and molecular results reveal that the Wang Khrachae samples differ from *C.auriventralis* and other known species of the *C.siamensis* group. We herein described this population as a new species.

## ﻿Materials and methods

### ﻿Sampling

*Cnemaspis* specimens were collected from Wang Khrachae Subdistrict, Sai Yok District, Kanchanaburi Province, western Thailand in November 2022 (Fig. 1). Geographic coordinates and elevations were recorded using a Garmin GPSMAP 64s with WGS84 datum. Live animals and preserved specimens were photographed using a Nikon Z50 digital camera with an AF-S Micro Nikkor 60-mm f/2.8G ED lens and external flashes. Captured specimens were humanely euthanised using tricaine methanesulfonate (MS-222) (Simmons 2015). Liver tissues were immediately removed from euthanised individuals, preserved in 95% ethanol and stored at -20 °C for molecular analysis. Euthanised specimens were fixed in 10% formalin and later transferred to 70% ethanol for permanent storage. Specimens and tissues were deposited in the herpetological collection of the Zoological Museum, Kasetsart University, Thailand (**ZMKU**).

### ﻿DNA extraction and PCR amplification

We extracted genomic DNA from liver tissue of two individuals of *Cnemaspis* from Wang Khrachae Subdistrict, Sai Yok District, Kanchanaburi Province (Fig. 1, Suppl. material 1) using the DNeasy Blood and Tissue Kit (Qiagen, Germany) according to the manufacturer’s protocol. The mitochondrial NADH dehydrogenase subunit 2 gene (ND2) and its flanking tRNAs was amplified via a double-stranded polymerase chain reaction (PCR) using the light strand primer L4437b (5’-AAGCAGTTGGGCCCATACC-3’; Macey et al. 1997) and heavy strand primer H5934 (5’ AGRGTGCCAATGTCTTTGTGRTT-3’; Macey et al. 1997). PCR reactions were executed in an Eppendorf Mastercycler gradient thermocycler under the following conditions: initial denaturation at 95 °C for 2 min, followed by a second denaturation at 95 °C for 35 s, annealing at 55 °C for 35 s, followed by a cycle extension at 72 °C for 35 s, for 33–40 cycles with a final extension at 72 °C for 10 min. PCR products were purified using a QIAquick PCR Purification Kit (Qiagen, Germany). PCR products were sequenced in both forward and reverse directions using the same amplifying primers at Bio Basic Asia Pacific PTe Ltd (Singapore) on an ABI 3730XL automatic sequencer (Applied Biosystems, CA, USA). Sequences were visually checked and edited in Geneious Prime 2021.0.3 (https://www.geneious.com). The protein-coding region of ND2 was translated to amino acids and checked to confirm the lack of premature stop codons. All new sequences were deposited in GenBank under accession numbers PQ659222–PQ659223 (Suppl. material 1).

### ﻿Phylogenetic analyses

Additional homologous sequences of 80 individuals belonging to the *C.affinis* group, *C.boulengeri* group, *C.argus* group, *C.chanthaburiensis* group, *C.kumpoli* group (= Pattani clade in Grismer et al. 2014), *C.siamensis* group and outgroups were downloaded from GenBank. *Cyrtodactylusbokorensis* Murdoch, Grismer, Wood, Neang, Poyarkov, Tri, Nazarov, Aowphol, Pauwels, Nguyen & Grismer, 2019 and *Hemidactylusgarnotii* Duméril & Bibron, 1836 were selected as outgroups to root the tree following Ampai et al. (2022) and Rujirawan et al. (2022). The two newly generated and downloaded *Cnemaspis* sequences were aligned using MAFFT v.7 online service (https://mafft.cbrc.jp/alignment/server/index.html; Katoh and Standley 2013). The aligned dataset was partitioned into four partitions consisting of 1^st^–3^rd^ND2 codon positions and tRNAs. Best-fit models of evolution for each partition were determined with ModelFinder (Kalyaanamoorthy et al. 2017) using the Bayesian information criterion (BIC). The best-fit evolutionary models were TPM2u+F+I+G4 for tRNAs and TVM+F+I+G4, TIM3+F+I+G4 and GTR+F+G4 for ND2 codon positions 1, 2 and 3, respectively.

Phylogenetic relationships were inferred through Maximum Likelihood (ML) and Bayesian Inference (BI). The ML analysis was performed using the IQ-TREE webserver 1.6.12 (Trifinopoulos et al. 2016) with 1,000 bootstrap pseudo-replicates using the ultrafast bootstrap analysis (Minh et al. 2013; Hoang et al. 2018). The BI analysis was implemented in MrBayes v.3.2 (Ronquist et al. 2012) on the CIPRES Science Gateway V. 3.3 (Miller et al. 2010) using default priors and models of evolution that were selected by ModelFinder and used in the ML analysis. Two independent runs, each with three heated and one cold chain, were performed using Markov Chain Monte Carlo (MCMC). The MCMC chains were run for 10,000,000 generations and trees sampled every 1,000 generations with the first 25% of each run discarded as burn-in. Stationarity was evaluated by ensuring effective sample sizes (ESS) were above 200 for all parameters in Tracer v. 1.7 (Rambaut et al. 2018). The phylogenetic trees from the ML and BI analyses were visualised using FigTree v. 1.4.4 (http://tree.bio.ed.ac.uk/software/figtree/). Nodes having ultrafast bootstrap support values (UFB) ≥ 95 and Bayesian posterior probabilities (BPP) ≥ 0.95 were considered well-supported (Huelsenbeck and Ronquist 2001; Wilcox et al. 2002; Minh et al. 2013). Uncorrected pairwise sequence divergences (*p*-distances) were calculated in MEGA 11 (Tamura et al. 2021) using the pairwise deletion option to remove gaps and missing data from the alignment prior to analysis.

### ﻿Morphology

Morphological measurements were taken with digital calipers to the nearest 0.1 mm. Scalation and other aspects of external morphology were examined using a Nikon SMZ745 stereomicroscope. Measurements were taken on the left side of the body, while scale counts were taken on both right and left sides (R/L) when possible. Morphological characters (measurements and meristics) and their abbreviations used follows Rujirawan et al. (2022): snout–vent length (**SVL**), taken from tip of snout to the anterior margin of vent ; tail width (**TW**) at the base of the tail immediately posterior to the postcloacal swelling ; tail length (**TL**), as distance from the vent to the tip of the tail, whether original, broken or regenerated ; forearm length (**FL**), taken on the dorsal surface from the posterior margin of the elbow while flexed 90° to the inflection of the flexed wrist ; tibia length (**TBL**), taken on the ventral surface from the posterior surface of the knee while flexed 90° to the base of the heel ; head length (**HL**), as distance from the posterior margin of the retroarticular process of the lower jaw to the tip of the snout ; head width (**HW**) at the angle of the jaws ; head depth (**HD**), as the maximum height of head from the occiput to the throat ; axilla-groin length (**AG**), taken from the posterior margin of the forelimb at its insertion point on the body to the anterior margin of the hind-limb at its insertion point on the body ; eye diameter (**ED**), as the maximum horizontal diameter of the eyeball ; eye-ear distance (**EE**), measured from the anterior margin of the ear opening to the posterior edge of the eyeball ; ear length (**EL**), taken from the greatest vertical distance of the ear opening ; eye-nostril distance (**EN**), measured from the anterior most margin of the eyeball to the posterior margin of the external nares ; eye-snout distance (**ES**), measured from the anterior margin of the eyeball to the tip of snout ; inner orbital distance (**IO**), as the width of the frontal bone at the level of the anterior edges of the orbit ; internarial distance (**IN**), measured between the medial margins of the nares across the rostrum ; supralabial scales (**SL**), counted from below the middle of the orbit to the rostral scale ; infralabial scales (**IL**), counted from below the middle of the orbit to the mental scale ; the number of paravertebral tubercles between limb insertions (**PVT**), counted in a straight line immediately left of the vertebral column ; the number of subdigital lamellae beneath the fourth toe (**4TL**), counted from the base of the first phalanx to the claw ; the total number of pore-bearing precloacal scales (**PP**) in males ; and the number of postcloacal tubercles (**PCT**) on each side of tail base. Additional character states evaluated were the general size (i.e. strong, moderate, weak) and arrangement (i.e. random or linear) of the dorsal body tubercles; the orientation and shape of precloacal pores; the number of precloacal scales lacking pores separating the left and right series of pore-bearing precloacal scales; the degree and arrangement of body and tail tuberculation; the relative size and morphology of the subcaudal scales, subtibial scales and submetatarsal scales beneath the first metatarsal. Sex and maturity were determined by the presence of secondary sexual characteristics, such as the presence of hemipenes or pore-bearing precloacal scales in males, the presence of eggs in females or sexually dimorphic colour patterns. Morphological data for comparisons were obtained from the original and expanded descriptions of other species in the *C.siamensis* group (Smith 1925; Grismer and Chan 2010; Grismer et al. 2010, 2014, 2020; Wood et al. 2017; Ampai et al. 2019, 2020, 2022; Rujirawan et al. 2022).

### ﻿Statistical analysis

All statistical analyses were conducted using R program v. 4.3.2 (R Core Team 2021). Morphophospatial clustering and positioning among species was analysed using principal component analysis (PCA) in the FactoMineR and factoextra packages (Lê et al. 2008; Kassambara and Mundt 2020). Specimens of three closely related species from Kanchanaburi Province were assigned to each group (= lineages, based on their mtDNA below), including, *C.auriventralis* (*n* = 5), *C.huaseesom* (*n* = 3) and the Wang Khrachae population (*n* = 2). For meristic characters, data were taken from the left side of examined specimens. Prior to the PCA analysis, fifteen morphometric characters (SVL, TW, FL, TBL, HL, HW, HD, AG, ED, EE, EL, EN, ES, IO, and IN) and four meristic characters (SL, IL, PVT, and 4TL) were concatenated into a single dataset. Precloacal pore (PP) was excluded from analyses due to their presence only in males. Tail length (TL) was excluded due to their different conditions (e.g., original, regenerated or broken). Postcloacal tubercle (PCT) was omitted due to inadequate data from *C.huaseesom*. To remove the effects of allometry, morphometric data (except SVL) were corrected for body-size variation using allometric growth model in the R package GroupStruct (available at http://github.com/chankinonn/GroupStruct) (Chan and Grismer 2022). Accordingly, the allometric formula is Xadj = log_10_(X) – ß[log_10_(SVL) – log_10_(SVLmean)], where Xadj = adjusted value; X = measured value; ß = unstandardized regression coefficient for each population and SVLmean = overall average SVL of all three populations (Thorpe 1975, 1983; Turan 1999; Lleonart et al. 2000).

## ﻿Results

### ﻿Phylogenetics

**Figure 2.**
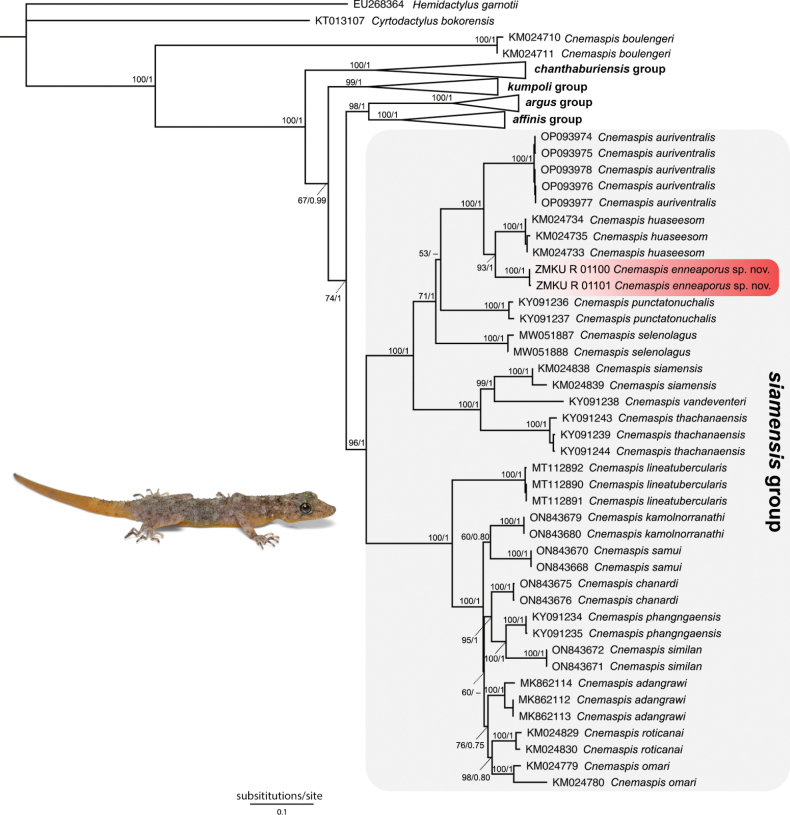
The best tree resulting from Maximum Likelihood analysis of 1,329 aligned characters of the mitochondrial NADH dehydrogenase subunit 2 gene and flanking tRNAs of *Cnemaspis* species. Nodal support is indicated by Ultrafast bootstrap (UFB) values and Bayesian posterior probabilities (BPP) from a separate Bayesian Inference analysis, respectively. GenBank accession numbers and locality data for sequenced samples are provided in Suppl. material 1.

The final alignment of ND2 and flanking tRNAs contained 1,329 characters of 82 individuals of *Cnemaspis* and the outgroups. The ML and BI analyses recovered trees with topologies similar to each other and to those recovered by Ampai et al. (2022) and Rujirawan et al. (2022) (Fig. 2). The two samples from Wang Khrachae formed a well-supported monophyletic lineage (100 UFB, 1.00 BPP) and embed within the *C.siamensis* group. The Wang Khrachae population was recovered as the sister taxon (93 UFB, 1.00 BPP) to *C.huaseesom* from Sai Yok National Park, Sai Yok District, Kanchanaburi Province. Both lineages, the Wang Khrachae and *C.huaseesom* lineages, formed a clade as the sister taxon to *C.auriventralis* from Erawan National Park, Si Sawat District, Kanchanaburi Province. Uncorrected pairwise genetic divergence (*p*-distances) between the Wang Khrachae samples was 0.2%. The Wang Khrachae population had uncorrected *p*-distances of 8.3–9.4% from *C.huaseesom* and 11.3–24.5% from the remaining species of the *siamensis* group (Suppl. material 2). Uncorrected *p*-distances divergences within the *siamensis* group ranged from 6.8% (between *C.adangrawi* and *C.omari*) to 27.5% (between *C.roticanai* and *C.vandeventeri*).

**Figure 3.**
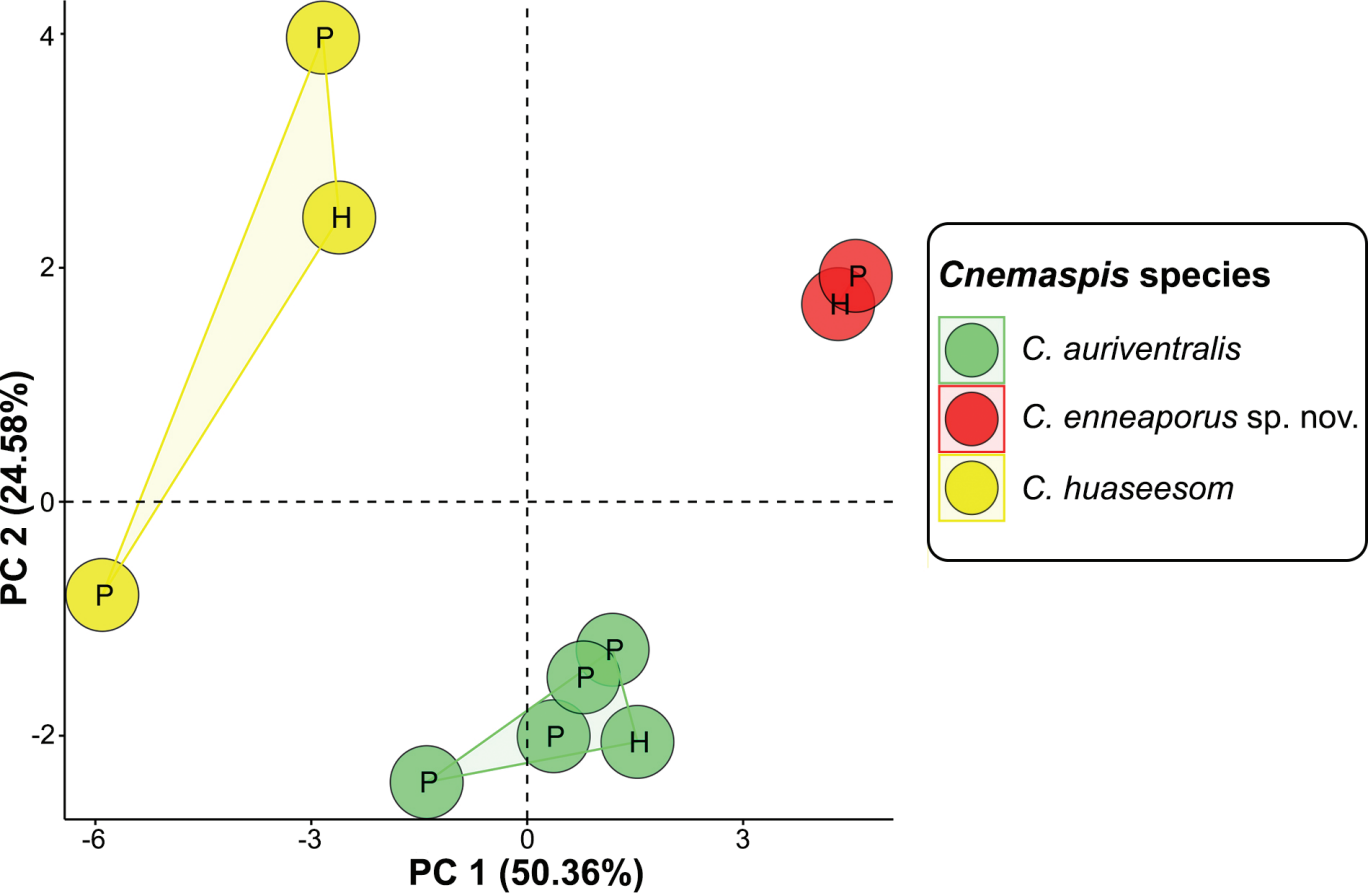
Plots of the first two principal components of *Cnemaspisenneaporus* sp. nov. and the closely related species based on 19 morphological characters. The letters in the scatter plots refer to holotype (H) and paratype (P).

The PCA analysis of three *Cnemaspis* lineages (the Wang Khrachae population, *C.auriventralis*, and *C.huaseesom*) from Kanchanaburi Province recovered morphological differences on a scatter plot of the first two PC axes (PC 1 and PC 2; Fig. 3). The first two axes accounted for 74.93% of cumulative of the total variation (50.36% for PC 1 axis and 24.58% for PC 2 axis; Table 1, Fig. 3). The factor loading of PC 1 was heavily loaded on most characters except HL and 4TL. The factor loading of PC 2 was heavily loaded on SVL, TW, TBL, HD, EN, ES, SL, PVT, and 4TL (Table 1). The PCA plot (first two axes) showed that the Wang Khrachae population is distinctly separated from *C.auriventralis* and *C.huaseesom* (Fig. 3). The *C.auriventralis* and *C.huaseesom* are also distinctly separated from each other.

**Table 1.** Factor loadings on the first five principal components (PC) of 19 morphological characters of *Cnemaspisenneaporus* sp. nov. and its closely related species, *C.auriventralis* and *C.huaseesom*. Morphological abbreviations are defined in Materials and methods.

Character	PC 1	PC 2	PC 3	PC 4	PC 5
SVL	0.8127	0.5248	0.08491	-0.08302	0.14543
TW	-0.5075	0.6983	-0.31075	0.24827	0.21863
FL	0.9096	0.3650	0.05729	-0.02468	0.04545
TBL	0.6322	-0.6381	-0.21347	-0.25227	0.14139
HL	0.2871	0.1055	0.90176	0.01232	-0.19016
HW	0.9572	-0.2027	-0.07465	-0.15252	0.00422
HD	0.7554	0.5998	-0.09544	0.09175	-0.04735
AG	0.9229	0.2483	-0.07096	0.06184	-0.15353
ED	0.7294	-0.0005	0.36329	-0.06324	0.50400
EE	0.6539	0.2684	-0.15147	-0.61508	-0.30723
EL	0.6080	0.2006	-0.23704	0.49270	-0.43409
EN	0.6812	0.6872	-0.01969	0.13057	0.19251
ES	0.6186	0.6850	-0.04838	0.02936	0.03754
IO	0.8802	-0.4025	-0.07851	0.00329	-0.15277
IN	0.9840	-0.0971	0.06670	-0.02101	0.03264
SL	0.5076	-0.7420	-0.27645	0.08171	0.08883
IL	0.5958	-0.4744	0.12948	0.60831	0.06261
PVT	-0.5957	0.5864	0.39654	-0.03285	-0.13491
4TL	-0.3375	0.7396	-0.38722	-0.08137	0.08159
Eigenvalue	9.568	4.669	1.610	1.196	0.789
Variance (%)	50.357	24.576	8.476	6.296	4.155
Cumulative variance (%)	50.357	74.933	83.409	89.705	93.860

### ﻿Taxonomic hypotheses

The *Cnemaspis* samples from Wang Khrachae Subdistrict, Sai Yok District, Kanchanaburi Province differed from congeners in mtDNA analyses, multivariate analysis, and diagnostic morphological characters (see “Comparisons”). Based on these corroborating lines of evidence, we hypothesize that the Wang Khrachae population represents a previously unnamed species, which is described below.

### ﻿Systematics

#### ﻿ Cnemaspis enneaporus

Taxon classificationAnimaliaSquamataGekkonidae

Rujirawan, Aksornneam & Aowphol sp. nov.

9A67AE1B-A1A0-55B2-8B6B-EA350C541DA5

https://zoobank.org/7B95FC67-EC87-46F6-80D7-8062350F4917

Figs 4 , 5 , 6 , 7

##### Type material.

**Figure 4.**
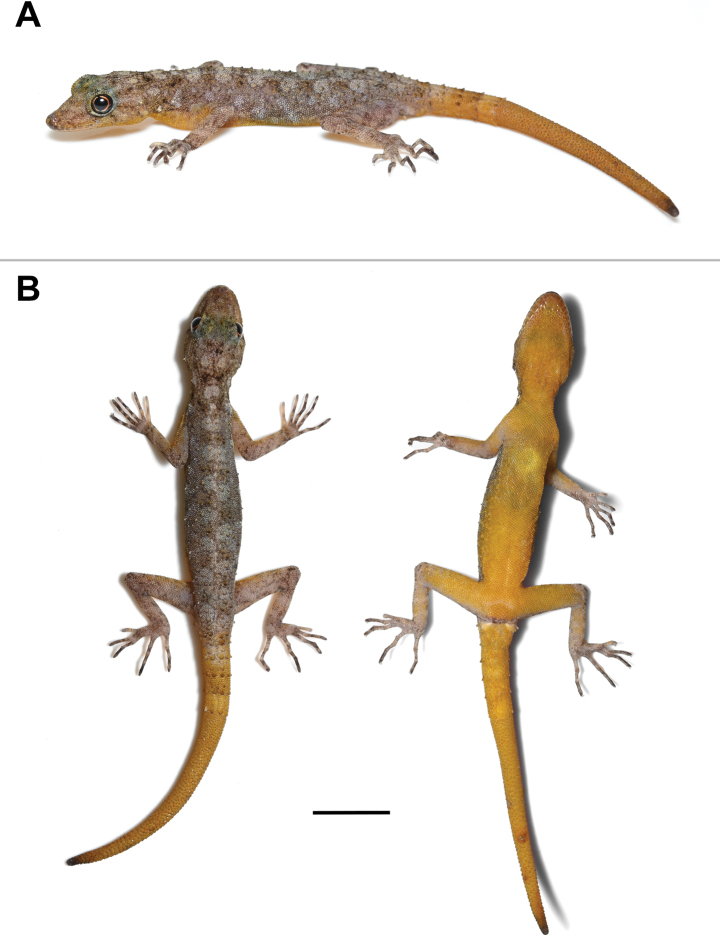
Adult male holotype of *Cnemaspisenneaporus* sp. nov. (ZMKU R 01100). **A** dorsolateral view in life **B** dorsal and ventral views immediately after euthanasia. Scale bar: 10 mm.

**Figure 5.**
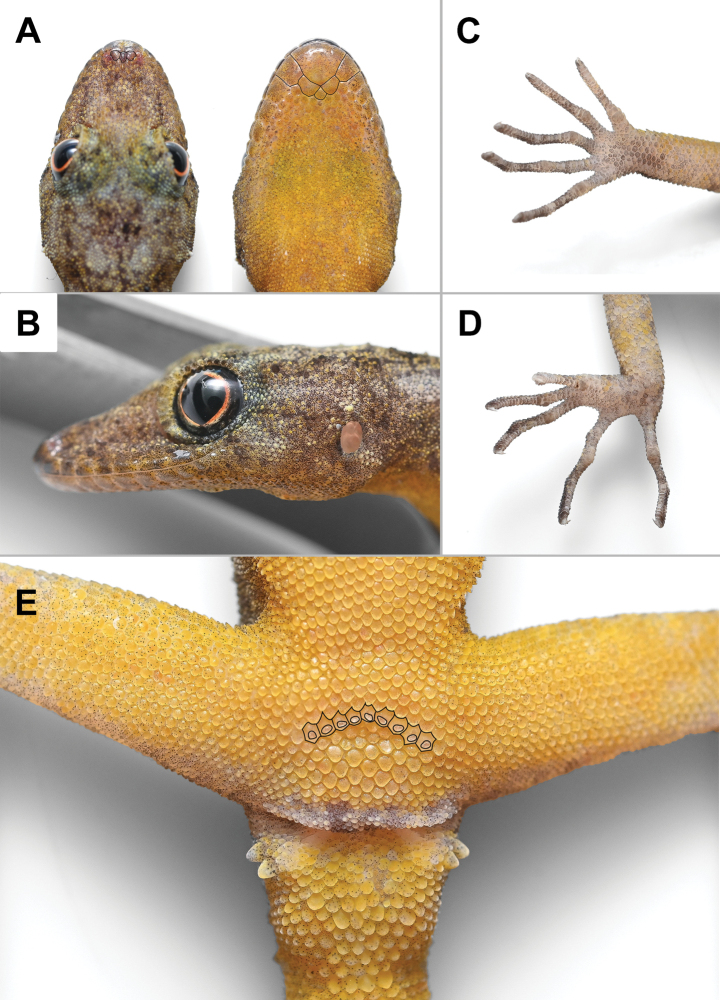
Adult male holotype of *Cnemaspisenneaporus* sp. nov. (ZMKU R 01100) immediately after euthanasia. **A** dorsal (supranasal and internasal scales outlined in black) and ventral views of head (mental, postmental, and first infralabial scales outlined in black) **B** lateral view of head **C** palmar view of the right manus **D** plantar view of the right pes **E** precloacal region with precloacal pores (outlined in black).

**Figure 6.**
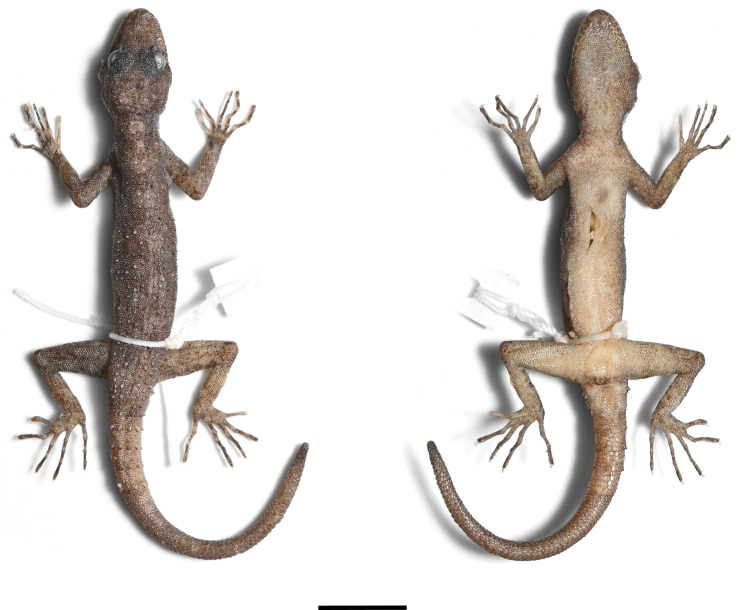
Dorsal and ventral views of adult male holotype of *Cnemaspisenneaporus* sp. nov. (ZMKU R 01100) in preservative. Scale bar: 10 mm.

**Figure 7.**
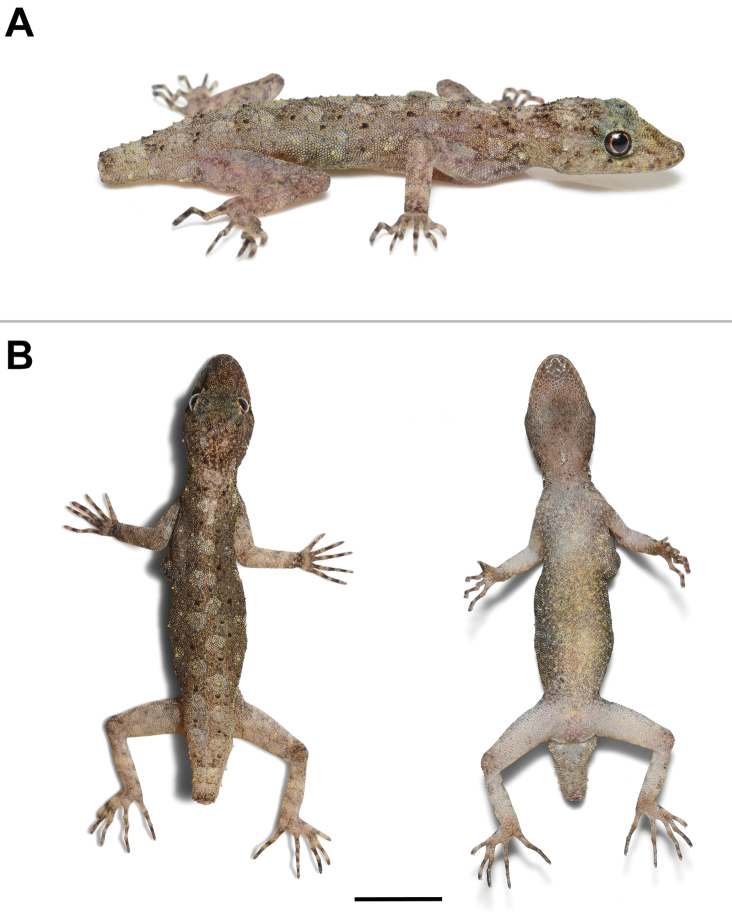
Adult female paratype of *Cnemaspisenneaporus* sp. nov. (ZMKU R 01101). **A** dorsolateral view in life **B** dorsal and ventral views immediately after euthanasia. Scale bar: 10 mm.

***Holotype*** (Figs 4–6). ZMKU R 01100, adult male from Thailand, Kanchanaburi Province, Sai Yok District, Wang Khrachae Subdistrict, (14.20247°N, 99.02222°E; 404 m elevation), collected on 18 November 2022 by Akrachai Aksornneam and Parinya Pawangkhanant. ***Paratype*** (Fig. 7). ZMKU R 01101, adult female, same collection data as the holotype.

##### Diagnosis.

*Cnemaspisenneaporus* sp. nov. can be distinguished from all other species in the *C.siamensis* group by having the unique combination of the following characters: SVL 42.2 mm in adult males (*n* = 1), 43.7 mm in adult females (*n* = 1); eight supralabials; seven or eight infralabials; ventral scales smooth; nine continuous precloacal pores in single male specimen; 17 or 18 paravertebral tubercles linearly arranged; tubercles on lower flanks present; lateral caudal furrows present; no caudal tubercles in the lateral furrows; ventrolateral caudal tubercles present on original portion of tail; caudal tubercles not encircling tail; subcaudals smooth; no enlarged median subcaudal row; two postcloacal tubercles on each side of tail; no shield-like subtibial scales; subtibial scales smooth; no enlarged submetatarsal scales; 26–28 subdigital lamellae on the fourth toe; sexually dimorphic for dorsal and ventral colour pattern; prescapular marking absent; gular marking absent; and yellow colouration in life on all ventral surfaces of head, body and tail in adult male.

##### Description of holotype.

Adult male; SVL 42.2 mm; head oblong in dorsal profile, moderate in size (HL/SVL 0.29), somewhat narrow (HW/SVL 0.18), flat (HD/HL 0.38), distinct from neck; snout moderate (ES/HL 0.43), snout slightly concave in lateral profile; postnasal region concave medially; scales of rostrum round, smooth, raised, larger than similarly-shaped scales on occiput; weak supraorbital ridges; weak frontorostral sulcus; canthus rostralis smoothly rounded; eye large (ED/HL 0.21); extra-brillar fringe scales small in general, but larger anteriorly; pupil round; ear opening oval, taller than wide; rostral concave dorsally, dorsal 50% divided by longitudinal groove; rostral bordered posteriorly by supranasals, one large azygous internasal and nostrils; bordered laterally by first supralabials; 8R/8L (right/left) raised supralabials of similar size, but smallest posteriorly; 7R/8L infralabials, decreasing gradually in size posteriorly; nostrils small, elliptical, orientated dorsoposteriorly; bordered posteriorly by two flat postnasal scales; mental large, triangular, flat, extending to level of second infralabials, bordered posteriorly by two postmentals; mental slightly concave; gular scales smooth, flat, round or oval, juxtaposed; throat scales round, smooth, raised, juxtaposed to subimbricate.

Body slender, elongate (AG/SVL 0.44); small, raised, weakly keeled, dorsal scales generally equal in size throughout body, intermixed with numerous, large, multi-keeled, linearly arranged tubercles; enlarged, multi-keeled, conical tubercles on flanks; tubercles extend from the occiput to base of the tail and continue on tail in whorls; body tubercles slightly smaller anteriorly; 18 paravertebral tubercles; pectoral and abdominal scales smooth, flat, imbricate; abdominal scales larger than pectoral and dorsal scales; nine contiguous, pore-bearing, precloacal scales; precloacal pores round to elongate.

Forelimbs moderately long, slender; dorsal scales raised, keeled, juxtaposed to subimbricate; ventral scales of brachia smooth, raised, juxtaposed to subimbricate; scales beneath forearm smooth, raised, subimbricate; digits long with an inflected joint; claws recurved; subdigital lamellae unnotched; subdigital lamellae wide throughout length of digits, bearing a larger scale at digital inflections; interdigital webbing absent; fingers increase in length from first to fifth, with fourth and fifth nearly equal in length; relative length of fingers I < II < III < V ≤ IV; total subdigital lamellae on fingers I–V: 14–19–25–27–26 (right), 14–20–24–26–broken (left). Hind-limbs slightly longer and thicker than forelimbs; dorsal scales keeled, raised, juxtaposed; ventral scales of thigh and subtibial scales smooth, flat, imbricate; plantar scales smooth, slightly raised, subimbricate; enlarged submetatarsal scales beneath first toes absent; digits elongate with an inflected joint; claws recurved; subdigital lamellae unnotched; lamellae wide throughout length of digits; enlarged scales at digital inflections; interdigital webbing absent; toes increase in length from first to fourth and fifth nearly equal in length; relative length of toes I < II < III < V ≤ IV; total subdigital lamellae on toes I–V: 13–18–23–28–25 (right), 13–18–26–28–26 (left).

Tail regenerated, long, slender, 42.1 mm in length (TL/SVL 1.00), tapering, becoming slender toward the tip; dorsal scales of the original portion of tail slightly keeled, raised, juxtaposed, arranged in segmented whorls; mid-dorsal and lateral, caudal furrows present; subcaudals smooth, flat, imbricate; median row of enlarged subcaudal scales absent; paravertebral, dorsolateral and lateral rows of large, keeled, caudal tubercles extend length of original tail; ventrolateral rows of tubercles extend length of original tail; caudal tubercle rows do not encircle tail; tubercles absent from lateral caudal furrow; scale of the regenerated portion of tail smooth, flat, imbricate; enlarged postcloacal tubercles 2R/2L on lateral surface of hemipenial swellings at base of tail.

##### Colouration in life

(Figs 4, 5). Dorsal ground colour of head, nape, trunk and limbs grey; dorsal ground colour of original and regenerate tail yellow; rostrum and interorbit regions bearing diffuse, faint, yellowish and brownish marking; top of head bearing small, diffuse, faint, dark, yellowish and light markings; dark postorbital stripes faint extending to occiput; pair of dark, diffuse, blotches on nape; large, light, irregularly-shaped, vertebral blotches extend from nape to base of tail, continuing on to original portion of tail as light yellow caudal bands; small, light, irregularly-shaped blotches in shoulder regions and flanks; limbs mottled with small, diffuse, dark marking; digits grey bearing dark bands. All ventral surfaces of head, body, thigh and original and regenerate portion of tail yellow; ventral surfaces of forelimbs and tibial region light grey with yellow speckling.

##### Colouration in preservative

(Fig. 6). Dorsal and lateral surfaces of head, body, limbs and tail darker grey than in life, with some fading of markings. Ventral surfaces of head, body, limbs and tail creamy-white, with minute dark speckling on gular region, limbs and tail regions; regenerate portion of tail darker than original tail.

##### Variation.

Variation in morphometric and meristic data between the holotype and paratype are presented in Table 2. *Cnemaspisenneaporus* sp. nov. shows significant sexual dimorphism in colour pattern. Yellow colouration on ventral surface of female (ZMKU R 01101) absent. Tail of the paratype is broken (TL = 6.3 mm) without yellow colouration of dorsal and ventral surface. The female paratype lack precloacal pores and has postcloacal tubercles that are relatively smaller than those in the male. Mental scale of the paratype bordered posteriorly by three postmental scales. The paratype has skin swelling on left side of axillary area.

**Table 2.** Descriptive measurements (mm) and meristics (right/left) of the type series of *Cnemaspisenneaporus* sp. nov. Morphological abbreviations are defined in Materials and Methods.

Museum number	ZMKU R 01100	ZMKU R 01101
**Type**	**holotype**	**paratype**
**Sex**	**male**	**female**
SVL	42.2	43.7
TW	3.2	3.7
TL	42.1	6.3
Tail condition	regenerated	broken
FL	7.0	7.4
TBL	7.5	7.6
HL	12.2	11.5
HW	7.6	7.6
HD	4.6	4.7
AG	18.7	20.0
ED	2.6	2.3
EE	3.0	3.0
EL	1.3	1.3
EN	4.3	4.3
ES	5.2	5.2
IO	2.8	2.8
IN	1.3	1.2
HL/SVL	0.29	0.26
HW/SVL	0.18	0.17
HD/HL	0.38	0.41
ES/HL	0.43	0.45
ED/HL	0.21	0.20
AG/SVL	0.44	0.46
TL/SVL	1.00	0.14
**Scalation**		
SL	8R / 8L	8R / 8L
IL	7R / 8L	7R / 8L
PVT	18	17
4TL	28R /28L	27R / 26L
PP	9 continuous	absent
PCT	2R / 2L	2R / 2L

##### Distribution and natural history.

**Figure 8.**
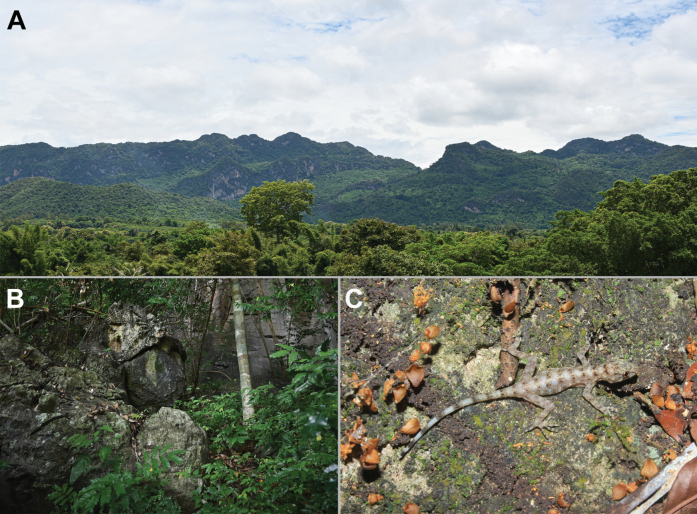
Habitat of *Cnemaspisenneaporus* sp. nov. at the type locality, Wang Khrachae Subdistrict, Sai Yok District, Kanchanaburi Province, Thailand. **A** landscape view of karst formation **B** karst microhabitat structure **C** adult female of *Cnemaspisenneaporus* sp. nov. (not collected) photographed *in situ* at the type locality.

The new species is known only from the type locality (Wang Khrachae Subdistrict, Sai Yok District, Kanchanaburi Province, Thailand) at ~ 400 m elevation in a karst formation that is part of the Tenasserim Mountain Range in western Thailand (Figs 1, 8). The holotype and paratype were found on karst boulders (~ 1.2–1.5 m height from ground) at night (00.00–00.30 h). One adult female (not collected; Fig. 8C) of *C.enneaporus* sp. nov. was found at the type locality on 31 August 2015 and was observed on a vine on a nearby karst wall (~ 1.5 m height from ground) at night (23.00 h). Other species of reptiles observed in the type locality were *Cyrtodactylussaiyok* Panitvong, Sumontha, Tunprasert & Pauwels, 2014, *Cyrtodactylustigroides* Bauer, Sumontha & Pauwels, 2003, *Gehyramutilata* (Wiegmann, 1834), *Dixoniushangseesom* Bauer, Sumontha, Grossmann, Pauwels & Vogel, 2004, and *Trimeresuruskanburiensis* Smith, 1943.

##### Etymology.

The species epithet *enneaporus* is derived from *ennea* (G.) for nine and *porus* (L.) for pore in reference to the male holotype of the new species having nine precloacal pores.

##### Suggested common name.

Sai Yok Rock Gecko (English), Jing Jok Niew Yao Sai Yok (Thai).

##### Comparisons.

*Cnemaspisenneaporus* sp. nov. is distinguishable from all other members of the *C.siamensis* group by a unique combination of morphological and colour pattern characteristics. *Cnemaspisenneaporus* sp. nov. differs from *C.adangrawi* Ampai, Rujirawan, Wood, Stuart & Aowphol, 2019 by having eight supralabials (vs 10); seven or eight infralabials (vs nine); ventral scales smooth (vs keeled); nine precloacal pores in male (vs 6–8 pores); precloacal pores continuous (vs separated); 17 or 18 paravertebral tubercles (vs 23–25 tubercles); paravertebral tubercles linearly arranged (vs randomly); tubercles on lower flanks present (vs absent); caudal tubercles in lateral furrow absent (vs present); subcaudal scales smooth (vs keeled); two postcloacal tubercles on each side in male (vs single tubercle); subtibial scales smooth (vs keeled); sexual dimorphism of dorsal colour pattern present (vs absent); light or yellowish prescapular crescent absent (vs present); yellow colouration on original and regenerated tail in male present (vs absent); and yellow colouration on all ventral surfaces of head, body and tail in male (vs yellowish colouration only on gular region, abdominal region and caudal region).

*Cnemaspisenneaporus* sp. nov. differs from *C.auriventralis* by having a larger maximum SVL of 43.7 mm (vs 38.6 mm); nine precloacal pores in male (vs 6 or 7 pores); and median row of subcaudal scales not enlarged (vs enlarged).

*Cnemaspisenneaporus* sp. nov. differs from *C.chanardi* Grismer, Sumontha, Cota, Grismer, Wood, Pauwels & Kunya, 2010 by having a larger maximum SVL of 43.7 mm (vs 40.1 mm); ventral scales smooth (vs keeled); nine precloacal pores in male (vs 6–8 pores); precloacal pores continuous (vs separated); 17 or 18 paravertebral tubercles (vs 22–25 tubercles); paravertebral tubercles linearly arranged (vs randomly); median row of subcaudal scales not enlarged (vs enlarged); subcaudal scales smooth (vs keeled); two postcloacal tubercles on each side in male (vs single tubercle); subtibial scales smooth (vs keeled); sexual dimorphism of dorsal colour pattern present (vs absent); light or yellowish prescapular crescent absent (vs present); yellow colouration on original and regenerated tail in male present (vs absent); and yellow colouration on all ventral surfaces of head, body and tail in male (vs yellow colouration only on gular region, belly, underside of hindlimbs and subcaudal region).

*Cnemaspisenneaporus* sp. nov. differs from *C.huaseesom* by having nine precloacal pores in male (vs 5–8 pores); caudal tubercles in lateral furrow absent (vs present); yellow colouration on dorsal surface of head and forelimbs in males absent (vs present); yellow colouration on regenerated tail in male present (vs absent); and yellow colouration on all ventral surfaces of head, body and tail in male (vs yellow colouration only on gular region, throat, pectoral region, underside of forelimbs and subcaudal region).

*Cnemaspisenneaporus* sp. nov. differs from *C.kamolnorranathi* Grismer, Sumontha, Cota, Grismer, Wood, Pauwels & Kunya, 2010 by having a larger maximum SVL of 43.7 mm (vs 37.8 mm); nine precloacal pores in male (vs seven pores); 17 or 18 paravertebral tubercles (vs 19–24 tubercles); caudal tubercles in lateral furrow absent (vs present); subcaudal scales smooth (vs keeled); sexual dimorphism of dorsal colour pattern present (vs absent); yellow colouration on original and regenerated tail in male present (vs absent); and yellow colouration on all ventral surfaces of head, body and tail in male (vs lacking yellow colouration on ventral surfaces).

*Cnemaspisenneaporus* sp. nov. differs from *C.lineatubercularis* Ampai, Wood, Stuart & Aowphol, 2020 by having ventral scales smooth (vs keeled); 17 or 18 paravertebral tubercles (vs 19–21); subcaudal scales smooth (vs keeled); two postcloacal tubercles on each side in male (vs single tubercle); subtibial scales smooth (vs keeled); sexual dimorphism of dorsal colour pattern present (vs absent); light or yellowish prescapular crescent absent (vs present); yellow colouration on original and regenerated tail in male present (vs absent); and yellow colouration on all ventral surfaces of head, body and tail in male (vs yellowish colouration only on anterior gular, abdominal and subcaudal regions).

*Cnemaspisenneaporus* sp. nov. differs from *C.omari* Grismer, Wood, Anuar, Riyanto, Ahmad, Muin, Sumontha, Grismer, Chan, Quah & Pauwels, 2014 by having ventral scales smooth (vs keeled); nine precloacal pores in male (vs 4 pores); precloacal pores continuous (vs separated); 17 or 18 paravertebral tubercles (vs 22–29 tubercles); caudal tubercles not encircling the tail (vs encircling); two postcloacal tubercles on each side in male (vs single tubercle); subtibial scales smooth (vs keeled); sexual dimorphism of dorsal colour pattern present (vs absent); light or yellowish prescapular crescent absent (vs present); yellow colouration on original and regenerated tail in male present (vs absent); and yellow colouration on all ventral surfaces of head, body and tail in male (vs yellow colouration only on gular region, belly, underside of hind-limbs, and subcaudal region).

*Cnemaspisenneaporus* sp. nov. differs from *C.phangngaensis* Wood, Grismer, Aowphol, Aguilar, Cota, Grismer, Murdoch & Sites, 2017 by having eight supralabials (vs 10); seven or eight infralabials (vs 10); ventral scales smooth (vs keeled); nine precloacal pores in male (vs 4 pores); 17 or 18 paravertebral tubercles (vs 22 tubercles); tubercles on lower flanks present (vs absent); subcaudal scales smooth (vs keeled); subtibial scales smooth (vs keeled); 26–28 subdigital lamellae on the fourth toe (vs 29 lamellae); light or yellowish prescapular crescent absent (vs present); yellow colouration on original and regenerated tail in male present (vs absent); and yellow colouration on all ventral surfaces of head, body and tail in male (vs yellow colouration only on anterior gular region, abdomen and subcaudal region).

*Cnemaspisenneaporus* sp. nov. differs from *C.punctatonuchalis* Grismer, Sumontha, Cota, Grismer, Wood, Pauwels & Kunya, 2010 by having a smaller maximum SVL of 43.7 mm (vs 49.6 mm); nine precloacal pores in male (vs absent); 17 or 18 paravertebral tubercles (vs 24–27); median row of subcaudal scales not enlarged (vs enlarged); 26–28 subdigital lamellae on the fourth toe (vs 29–31 lamellae); ocelli on brachium and side of neck in male absent (vs present); yellow colouration on original tail in male present (vs absent); and yellow colouration on all ventral surfaces of body and tail in male (vs orange colouration on throat and subcaudal region).

*Cnemaspisenneaporus* sp. nov. differs from *C.roticanai* Grismer & Chan, 2010 by having a smaller maximum SVL of 43.7 mm (vs 47.0 mm); ventral scales smooth (vs keeled); nine precloacal pores in male (vs 3–6 pores); 17 or 18 paravertebral tubercles (vs 25–27 tubercles); paravertebral tubercles linearly arranged (vs randomly); median row of subcaudal scales not enlarged (vs enlarged); subcaudal scales smooth (vs keeled); subtibial scales smooth (vs keeled); light or yellowish prescapular crescent absent (vs present); and yellow colouration on original tail in male present (vs absent).

*Cnemaspisenneaporus* sp. nov. differs from *C.samui* Ampai, Rujirawan, Yodthong, Termprayoon, Stuart, Wood & Aowphol, 2022 by having ventral scales smooth (vs keeled); nine precloacal pores in male (vs 5–8 pores); precloacal pores continuous (vs separated); 17 or 18 paravertebral tubercles (vs 25–27 tubercles); paravertebral tubercles linearly arranged (vs randomly); median row of subcaudal scales not enlarged (vs enlarged); subcaudal scales smooth (vs keeled); subtibial scales smooth (vs keeled); 26–28 subdigital lamellae on the fourth toe (vs 22–25 lamellae); sexual dimorphism of dorsal colour pattern present (vs absent); light or yellowish prescapular crescent absent (vs present); yellow colouration on original and regenerated tail in male present (vs absent); and yellow colouration on all ventral surfaces of head, body and tail in male (vs yellowish colouration only on gular region, abdomen, limbs and subcaudal region).

*Cnemaspisenneaporus* sp. nov. differs from *C.selenolagus* Grismer, Yushchenko, Pawangkhanant, Nazarov, Naiduangchan, Suwannapoom & Poyarkov, 2020 by having a larger maximum SVL of 43.7 mm (vs 36.2 mm); eight supralabials (vs 10–11); seven or eight infralabials (vs 10); nine precloacal pores in male (vs six or seven pores); paravertebral tubercles linearly arranged (vs randomly); tubercles on lower flanks present (vs absent); lateral caudal furrow present (vs absent); caudal tubercles not encircling the tail (vs encircling); enlarged submetatarsal scales on the first toe absent (vs present); 26–28 subdigital lamellae on the fourth toe (vs 22 lamellae); orange-yellow colouration on anterior 1/2 of body in male absent (vs present); ocelli on brachium and side of neck in males absent (vs present); light or yellowish prescapular crescent absent (vs present); yellow colouration on dorsal surface of forelimbs in male absent (vs present); yellow colouration on original and regenerated tail in male present (vs absent); and yellow colouration on all ventral surfaces of head, body and tail in male (vs yellow colouration only on anterior part of body).

*Cnemaspisenneaporus* sp. nov. differs from *C.siamensis* (Smith, 1925) by having a larger maximum SVL of 43.7 mm (vs 39.7 mm); ventral scales smooth (vs keeled); nine precloacal pores in male (vs absent); 17 or 18 paravertebral tubercles (vs 19–25 tubercles); paravertebral tubercles linearly arranged (vs randomly); median row of subcaudal scales not enlarged (vs enlarged); subcaudal scales smooth (vs keeled); subtibial scales smooth (vs keeled); sexual dimorphism of dorsal colour pattern present (vs absent); yellow colouration on original and regenerated tail in male present (vs absent); lineate gular marking absent (vs present); and yellow colouration on all ventral surfaces of head, body and tail in males (vs yellow colouration only on gular region, throat and pectoral region).

*Cnemaspisenneaporus* sp. nov. differs from *C.similan* Ampai, Rujirawan, Yodthong, Termprayoon, Stuart, Wood & Aowphol, 2022 by having a smaller maximum SVL of 43.7 mm (vs 48.1 mm); ventral scales smooth (vs keeled); nine precloacal pores in male (vs one pore); 17 or 18 paravertebral tubercles (vs 24 or 25 tubercles); paravertebral tubercles linearly arranged (vs randomly); subcaudal scales smooth (vs keeled); subtibial scales smooth (vs keeled); 26–28 subdigital lamellae on the fourth toe (vs 23 or 24 lamellae); light or yellowish prescapular crescent absent (vs present); yellow colouration on original and regenerated tail in male present (vs absent); and yellow colouration on all ventral surfaces of head, body and tail in male (vs pale yellowish blotches or reticulum on gular, neck, limbs and belly).

*Cnemaspisenneaporus* sp. nov. differs from *C.thachanaensis* Wood, Grismer, Aowphol, Aguilar, Cota, Grismer, Murdoch & Sites, 2017 by having a larger maximum SVL of 43.7 mm (vs 39.0 mm); eight supralabials (vs 10 or 11); seven or eight infralabials (vs nine or 11); ventral scales smooth (vs keeled); nine precloacal pores in male (vs absent); subcaudal scales smooth (vs keeled); two postcloacal tubercles on each side in male (vs absent); subtibial scales smooth (vs keeled); enlarged submetatarsal scales on the first toe absent (vs present); 26–28 subdigital lamellae on the fourth toe (vs 23–25 lamellae); yellow colouration on original and regenerated tail in male present (vs absent); lineate gular marking absent (vs present); and yellow colouration on all ventral surfaces of head, body and tail in male (vs yellowish-orange colouration only on gular region).

*Cnemaspisenneaporus* sp. nov. differs from *C.vandeventeri* Grismer, Sumontha, Cota, Grismer, Wood, Pauwels & Kunya, 2010 by having ventral scales smooth (vs keeled); nine precloacal pores in male (vs four pores); 17 or 18 paravertebral tubercles (vs 25–29 tubercles); paravertebral tubercles linearly arranged (vs randomly); tubercles on lower flanks present (vs absent); median row of subcaudal scales not enlarged (vs enlarged); subcaudal scales smooth (vs keeled); subtibial scales smooth (vs keeled); sexual dimorphism of dorsal colour pattern present (vs absent); light or yellowish prescapular crescent absent (vs present); yellow colouration on original and regenerated tail in male present (vs absent); and yellow colouration on all ventral surfaces of head, body and tail in male (vs orange colouration on gular region, throat, pectoral region, underside of limbs, belly and subcaudal region).

## ﻿Discussion

*Cnemaspisenneaporus* sp. nov. superficially resembles *C.auriventralis* from Erawan National Park in colouration pattern of adult male (yellow colouration on all ventral surface of head, body and subcaudal regions). However, the new species is closely related to *C.huaseesom* from Sai Yok National Park and these two species form the sister clade to *C.auriventralis* from Erawan National Park. These three species were found in similar habitat (karst-associated area) in Kanchanaburi Province, western Thailand. Moreover, the type locality of the new species is ca 20 km and 30 km from *C.auriventralis* and *C.huaseesom*, respectively. However, the combination of morphology, phylogenetic position and high genetic divergences (8.3–11.9%) revealed clear differences among them. Unfortunately, the new species is known from only two specimens (one individual was not collected; Fig. 8C) from the type locality at ~ 400 m elevation. During January 2018 and February 2019, Aksornneam et al. (2023) conducted ecological surveys of two sympatric geckos, *Cyrt.saiyok* and *Cyrt.tigroides* in the karst habitats within the type locality of *C.enneaporus* sp. nov. at elevation ranging from 250–350 m. The new species was not observed during their study or in previous surveys conducted below 350 m elevation (AAK and AR pers. obs.). This suggests that the geographic distribution of the new species could be limited by elevation (above 400 m). However, the highest elevation surveyed was ca 500 m (AAK pers. obs.), while the peak of the karst hill is ca 800 m. Additional surveys at higher and lower elevations are needed to better understand the ecological niche and microhabitats of the new species.

The description of *C.enneaporus* sp. nov. brings the total number of Thai *Cnemaspis* species to 24 (23 Southeast Asian clade members + 1 South Asian clade member) and the number of species in the *siamensis* group to 17 (Ampai et al. 2022; Rujirawan et al. 2022; Uetz et al. 2024). Karst or limestone areas are special and spectacular types of landscapes created by the dissolution of carbonate rocks, consisting of a diverse array of microhabitats shaped by complex terrains and variable climatic conditions (Clements et al. 2006; van Beynen 2011; Jantarit and Ellis 2023). In Thailand, karst landscape covers ~ 18% of the land area (ca 93,000 km^2^) and the largest karst area in the country (covering ca 12,00 km^2^) is in Kanchanaburi Province, western region (Sidisunthorn et al. 2006; Bolger and Ellis 2015; Jantarit and Ellis 2023). Previously, several herpetofauna species, which are apparently restricted to karst habitats, were discovered from Kanchanaburi Province i.e. *C.auriventralis*, *C.huaseesom*, *Cyrtodactylusmonilatus* Yodthong, Rujirawan, Stuart, Grismer, Aksornneam, Termprayoon, Ampai & Aowphol, 2022, *Cyrtodactylusfluvicavus* Grismer, Aowphol, Yodthong, Ampai, Termprayoon, Aksornneam & Rujirawan, 2022, *Cyrt.tigroides*, *D.hangseesom*, *Gekkonutaphandi* Bauer, Sumontha & Pauwels, 2008, *Oligodonsaiyok* Sumontha, Kunya, Dangsri & Pauwels, 2017, *Siamophrynetroglodytes* Suwannapoom, Sumontha, Tunprasert, Ruangsuwan, Pawangkhanant, Korost & Poyarkov, 2018, and *T.kanburiensis* (Smith 1943; Bauer et al. 2003, 2004, 2008; Sumontha et al. 2017; Suwannapoom et al. 2018; Grismer et al. 2022; Rujirawan et al. 2022; Yodthong et al. 2022). The discovery of the new species of *Cnemaspis* in association with karst underscores the significance of the karst habitats in Kanchanaburi for herpetofauna diversity and endemism within Thailand. It also highlights the need for additional field surveys in the karst formations along the northern part of the Tenasserim Mountain Range in western Thailand to determine the total geographic range of *C.enneaporus* sp. nov. and its closely relatives and enhance the documentation of herpetofauna diversity in Thailand.

## Supplementary Material

XML Treatment for Cnemaspis enneaporus

## ﻿Additional information

### Author ORCIDs

Attapol Rujirawan https://orcid.org/0000-0001-9179-6910

Akrachai Aksornneam https://orcid.org/0000-0003-4780-376X

Siriporn Yodthong https://orcid.org/0000-0001-6577-5358

Parinya Pawangkhanant https://orcid.org/0000-0002-0947-5729

Bryan L. Stuart https://orcid.org/0000-0003-4719-1951

Anchalee Aowphol https://orcid.org/0000-0001-9504-4601

### Data availability

All of the data that support the findings of this study are available in the main text or Supplementary Information.

## Supplementary materials

Supplementary material 1 Samples used in the molecular analyses, including their locality, voucher number and GenBank accession number Data type xlsx Explanation note Voucher abbreviations are the School of Agriculture and Natural Resources, University of Phayao (AUP), Monte L. Bean Life Science Museum at Brigham Young University (BYU), California Academy of Sciences (CAS), the Field Museum of Natural History, Chicago, Illinois, USA (FMNH), La Sierra University Herpetological Collection (LSUHC), Universiti Sains Malaysia Herpetological Collection at the Universiti Sains Malaysia, Penang, Malaysia (USMHC), Zoological Museum of Kasetsart University (ZMKU) and the Zoological Museum of Moscow University (ZMMU). https://binary.pensoft.net/file/1242984This dataset is made available under the Open Database License (http://opendatacommons.org/licenses/odbl/1.0/). The Open Database License (ODbL) is a license agreement intended to allow users to freely share, modify, and use this Dataset while maintaining this same freedom for others, provided that the original source and author(s) are credited.Attapol Rujirawan, Akrachai Aksornneam, Siriporn Yodthong, Parinya Pawangkhanant, Bryan L. Stuart, Anchalee Aowphol

Supplementary material 2 Mean percentages of uncorrected pairwise sequence divergences (*p*-distances) Data type xlsx Explanation note Mean (minimum-maximum) percentages of uncorrected pairwise sequence divergences (*p*-distances) of *Cnemaspis* species in the *C.siamensis* group compared to *Cnemaspisenneaporus* sp. nov., based on 1,329 aligned characters of the mitochondrial NADH dehydrogenase subunit 2 gene and flanking tRNAs. Intraspecific p-distances are in bold font. https://binary.pensoft.net/file/1242985This dataset is made available under the Open Database License (http://opendatacommons.org/licenses/odbl/1.0/). The Open Database License (ODbL) is a license agreement intended to allow users to freely share, modify, and use this Dataset while maintaining this same freedom for others, provided that the original source and author(s) are credited.Attapol Rujirawan, Akrachai Aksornneam, Siriporn Yodthong, Parinya Pawangkhanant, Bryan L. Stuart, Anchalee Aowphol
